# Reflex Action and Its Value in the Diagnosis of Spinal Diseases

**Published:** 1886-03

**Authors:** Edward B. Angell

**Affiliations:** Rochester, N. Y.


### *Reflex Action and its Value in the Diagnosis of Spinal Diseases.

By Edward B. Angell, M. D., Rochester, N. Y.

As a preface to our subject, it may be well to give a résumé of the grosser points in the anatomy and physiology of the spinal cord. Anatomically the cord is that portion of the cerebro-spinal axis lying in the vertebral canal. While in the fœtus and new-born child it extends the full length of the canal, in the adult it only reaches the lower border of the first or second lumbar vertebra, though the remaining extent of the canal is occupied in part by a slender filament of gray matter known as the terminal filament. As a consequence, the various pairs of nerves, except the highest, do not arise from the cord opposite the vertebræ whence they leave the canal and after which they are named, but at a higher level. This difference between the levels of origin and exit increases as we descend the cord, until in the canda equina the lowest nerves have a very long course from the end of the cord to their foramina. This relation, important frequently in the location of disease, is further complicated by the fact that the vertebral spines, which alone we can feel, and which therefore constitute our landmarks, do not correspond throughout the whole column to their vertebræ.

For the purposes of this paper, it is needless to take cognizance of the enveloping membranes of the spinal cord further than to state that essentially they form a continuation of similar membranes surrounding the brain.

If, now, we examine the general structure of a transverse section of the cord, we shall find the following characteristic features essentially the same throughout its whole extent. The section is divided into two halves by the anterior and posterior median fissures, the position of each being marked by a depression in the surface. These fissures do not wholly bisect

*Read before the Rochester meeting of the New York Central Medical Association.

the cord, owing to the interposition of a band of transverse fibres, through the center of which passes the small central canal, well marked in fœtal life, but usually in the adult obliterated by cell debris. Each lateral half of the cord is semi-cylindrical in form, the convex lateral surface of which is marked by a prominent depression—the postero-lateral fissure—which, in some portions of the cord, is as conspicuous as the antero-median fissure. It is designed to receive the round bundles of the posterior or sensory nerve-roots. This postero-lateral fissure divides the lateral half of the cord into two columns, the antero-lateral column lying between the antero-median fissure and the postero-lateral fissure, and the posterior column lying between the postero-lateral fissure and the postero-median fissure. These columns are continuous with corresponding columns in the medulla. In fact, the division of the medulla from the cord is purely arbitrary, since, in its physiological relations and functions, the cord extends from the sella turcica to the terminal filament. The surface of this transverse section we find on inspection is marked off into dark and white portions. The dark portion takes the form of a crescentic bar or nucleus in the center of the white portion of each half of the cord, and, with the posterior band or commissure of similar substance uniting them, constitutes the gray or cineritious matter. Each bar is rounded or widened in front, forming the anterior cornu. It is enclosed by white matter. The posterior end of the bar is pointed to form the posterior horn, or cornu, and is in part continuous with the postero-lateral fissure.

Of the minute anatomy of the spinal cord it will be needless to remark further than that the gray matter is composed of nerve cells and the white matter of nerve fibres, arranged in groups of longitudinal, transverse, and oblique filaments. The nerve cells are most conspicuous in the anterior horns, and are supposed to preside over the transformation of nerve excitation, while the nerve fibres, of which the longitudinal ones are most abundant, conduct nerve excitation.

A spinal nerve, tracing it backward from the periphery, is composed of sensory or afferent, and motor or efferent, fibres—the term sensory being used in a strictly physiological sense, and not involving the idea of sensation. Just within the intervertebral foramina the fibres become separated into two distinct bundles. The anterior one is made up of motor threads, which have their origin either in the protoplasmic processes from the cells of the anterior horn, or from the axis cylinders grouped about the cells. The posterior, or sensory bundle, has, just beyond this division, an enlargement known as the spinal ganglion, whose function is not clear. This bundle is composed of sensory fibres, which enter the postero-lateral fissure and disperse, tending to be deflected medianly through the posterior column. Their ultimate course is unknown.

The paths of reflex action to peripheral stimulation must be centripetally through the posterior nerve roots, and ordinarily by fine filaments of gray matter to the anterior nerve cells of the same side. Thence it passes centrifugally by the anterior motor nerves toward the periphery where the irritation was applied. In addition to this direct path, we have, within the cord, others of greater resistance; for, should our stimulus be of sufficient strength, the excitation will diffuse itself more widely and affect more nerve roots, as shown by the laws of reflex action. The higher cerebral centers are also connected with the reflex centers by paths of conduction, which are believed to lie in the lateral columns of white matter, through which is transmitted the normal inhibitory action that the cerebral centers seem to exercise over the reflex function of the spinal nerve ganglia. A classification of reflex actions, with reference to their course, must embrace not only the nerves of the cerebro-spinal system, but also those of the sympathetic. First, a spinal nerve trunk alone may constitute the course of a reflex excitation in both directions. This forms the most numerous class, and embraces such normal reflex acts as deglutition, coughing, sneezing, walking, etc.; and, in pathology, a large number of morbid reflex actions, as vomiting, tetanus, epilepsy, etc.

A second class, nearly as numerous, comprises those reflex actions, of which the centripetal direction is along the course of a sensory nerve of the cerebro-spinal system, while the centrifugal direction is along a motor nerve of the sympathetic, most often a vaso-motor nerve. Of this class are the reflex actions that give rise to most of the secretions, the saliva, gastric juice, etc.; to the phenomenon of blushing; to certain modifications in the pulsations of the heart, etc.; and, in pathology, to a large number of phenomena called metastatic. A third class includes those reflex actions whose centripetal direction is along a branch of the sympathetic, but whose centrifugal course is of a cerebro-spinal motor nerve. Most phenomena of this group belong to pathology, such as convulsions produced by intestinal worms, reflex eclampsia, etc. In the fourth class, far more obscure, are grouped those reflex actions, the course of which, in both directions, is the nerve fibres of the sympathetic system alone. Possibly to this class belongs that peculiar physiological sympathy existing between the uterus and breasts, with other obscure phenomena of the generative functions.

Above I referred to paths of conduction within the cord itself of different resistances, intimating that the concentration or diffusion of reflex action depended upon the energy of the excitation. This relation between the intensity of excitation and its reflex anatomical distribution was first established by Pflüger, and is known as Pflüger's laws of reflex action. These laws, perhaps, are best defined by illustrations. If, for instance, a sensory nerve of the lower extremity is excited by a feeble stimulus, a reflex motion is developed in the muscles of the same extremity; *i. e.*, in the muscles whose motor nerve supply starts from the spinal cord on the same side, and at the same height, as the sensory fibres thus stimulated—an illustration of the law of *unilaterality*. If, now, the excitation becomes more intense, the motory re-action is also manifested in the corresponding muscles of the opposite side—the law of *symmetry;* but always with less intensity than in those receiving the sensory stimulation—the law of *intensity*. Finally, if the excitation is still increased, the motor re-action is extended to motor fibres of a different height, but always upward toward a higher plane of the spinal cord—the law of *radiation*. If, however, the excitation and consequent motor re-action are sufficiently energetic to be propagated upward as far as the protuberance, the re-action becomes general and is manifested in every direction, both downward and upward, producing general muscular spasm—the law of *generalization*.

This power of transforming centripetal impressions into centrifugal activity, called reflex power, is the function of the gray axis of the spinal cord, and particularly of the nerve cells in the anterior horns. This reflex power is modified by the action of certain agents. It is increased by a high temperature; *i. e.*, reflex actions are more readily provoked in summer than in winter. It is also increased by a certain number of poisons; strychnine, morphine, picrotoxine, nicotine, veratrine, as well as by certain pathological products of the organism, such as we get in septicæmia, uræmia, and severe icterus. On the contrary, reflex power is diminished by the depressing effect of numerous successive irritations; by anæmia; by certain toxical agents, such as hydrocyanic acid, potassium bromide, atropine, calabar bean, chloral, nitrite of amyl, and other drugs of the depressor motor class.

Further, Setschenow has advanced the theory that there exists an inhibitory center controlling reflex action, located in the corpora quadrigemina and thalami optici. Many physiologists, however, dispute this; though it is certain that whenever the spinal cord is separated from the medulla by cross-section or disease of the conducting tracts, there exists an abnormal readiness to reflex action in those planes of the cord below the point of separation.

The value of the reflex activity in disease is, that its normal persistence is proof that there is no considerable disease in the reflex loops through which it is transmitted. Its absence or presence to an excessive degree is, in some cases, equally important.

It is necessary to distinguish two forms of reflex action—the skin reflexes and the tendon reflexes. Less importance is attached to the first method of testing reflex activity, but its value appears to be in pointing out the location of the injury rather than in determining such injury. But the tendon reflexes play a more important role, and often establish a diagnosis suggested by other symptoms.

Let us now examine the normal reflex actions and the means of demonstrating their presence, considering, first, the skin reflexes. If we stroke, tickle, or otherwise gently irritate the plantar surface of either foot, there follows a contraction of the muscles of the foot so irritated, the *plantar* reflex, and of the foot muscles alone, in accordance with law of unilaterality. The reflex loop thus stimulated includes the lower part of the lumbar enlargement. Again, irritation of the skin of the buttocks, in some individuals, excites a contraction of the glutis—the *gluteal* reflex—its course involving the cord at the level of the fourth or fifth lumbar nerves. Next, there is the well-known *cremaster* reflex, by which the testicle is drawn up when the skin on the inner surface of the thigh is stimulated. This arises at the level of the first and second lumbar pairs. This reflex is the readiest obtained of all, mere stroking of the skin usually producing it. It is even more marked if the adductor mass of the thigh is grasped quickly and firmly, and I have even seen the testicle of the opposite side drawn up as well, though to a less degree, neatly illustrating two laws of reflex action—those of symmetry and intensity. Then, there is the *abdominal* reflex, a contraction of the abdominal muscles, produced by stroking the skin on the side of the abdomen, from the edge of the ribs downward. This reflex loop is located in the cord, from the eighth to the twelfth dorsal nerves. Higher up, by stimulating the side of the chest in the sixth, fifth, and sometimes the fourth, intercostal spaces, we get an *epigastric* reflex, depending on contraction of the highest fibres of the rectus abdominis. It is related to the cord at the origin of the fourth, fifth, sixth or seventh pairs of dorsal nerves. There is no higher reflex on the front of the trunk, but if we test the back, we can, in some patients, get reflex contraction of the scapular muscles, sometimes very slight, sometimes quite marked. It is the highest skin reflex obtainable, and involves the cord at the level of the upper two or three dorsal, and the lower two or three cervical, nerves. Thus, by these reflexes—plantar, gluteal, cremasteric, abdominal, epigastric and scapular—we have means of ascertaining something of the condition of almost every inch of the spinal cord, from the cervical enlargement downward. Their presence is proof that the reflex path through the cord is not seriously interrupted, but we cannot simply infer, from their absence, that this path is impaired.

The second group of phenomena, of far more importance to the diagnostitian, includes those known as the "tendon reflexes." We will first consider the well-known jerk of the leg, which occurs when the patellar tendon is tapped. It has been called the "knee phenomenon" by Westphal, who first pointed it out, and the "patellar tendon reflex" by Erb. It is a little interesting that this knee jerk, well known to school-boys for generations, has only recently become established as of important clinical significance. To obtain the jerk, the knee must be flexed so that the quadratus femoris is gently extended and the leg free to move. Ordinarily this position can be obtained by simply crossing the leg to be tested over its fellow, allowing it to hang freely. But in many cases, especially if the individual be stout, it is preferable for the observer to support the thigh, just above the knee, with his own arm, resting the hand on the patient's other knee; and to have positive evidence that the reflex is deficient, the leg should be bared. If now, the leg in proper position, the tendon below the patella be gently tapped with the side of the extended hand, a muscular contraction, causing the foot to jerk upward, will be produced. We must carefully distinguish between the direct effect of the blow and the muscular contraction; and, when the contraction is slight, it may be better elicited by a percussion hammer or the edge of a stethoscope. Again, the same result may often be obtained by a downward blow upon the patella, or by a blow upon the quadriceps femoris above the patella.

The next important phenomenon of this group occurs at the ankle joint. If the calf muscles connected with the achilles tendon are made tense, and this tendon is tapped, the muscles contract, causing a slight extension movement of the foot, similar to the reflex movement at the knee. But under normal conditions this re-action is slight, and is only of importance in detecting increased irritability of the reflex path. But when these phenomena are excessive, if the calf muscles are suddenly made tense by pressing the hand against the sole of the foot; *i. e.*, by producing dorsal flexion, a quick contraction occurs; and, if the pressure be kept up, a clonic series of spasmodic contractions—the "ankle clonus" or "foot clonus"—is established. It can best be obtained when the leg is not comple'ely extended. The motion is very uniform, from six to eight contractions occurring per second. These two form the most important of the tendon reflexes. Yet whenever, owing to lateral degeneration of the cord high up, we have overaction of the reflex centers, we can get tendon reflexes elsewhere; for instance, in the peroneal tendons, the flexor tendons of the hand, and the biceps or triceps tendons of the arm. But it is the knee jerk and ankle clonus more especially that are of importance in the diagnosis of spinal maladies.

It is needless to enter into detail regarding the essential nature of these tendon reflexes further than to state, that at present they are believed to depend upon increased muscle tension; hence called myotatic contractions by Gowers, and are really reflex in their origin.

In testing the spinal reflexes, it is necessary to examine both sides of the body, since often the variation in activity between the corresponding reflex loops furnishes valuable and essential features in our diagnosis.

Let us now consider the character and location of the pathological processes determined by the presence or absence of these tendon reflexes. In departing from the normal reflex activity, we find that these myotatic contractions may be increased even to marked exaggeration, or diminished even to complete obliteration. An exaggeration of these re-actions must necessarily depend upon increased nerve irritability, while, on the contrary, a decrease or obliteration of the myotatic contractions must depend upon diminished irritability in the reflex path.

Remembering that a reflex loop is made up of sensory conducting fibres passing into the posterior root zone; thence, by delicate connecting fibres to the anterior motor nerve cells, whence the motor conducting fibres pass outward to the periphery; and, that in the lateral columns are found the conducting fibres, through which the inhibitory center in the brain exercises its restraint over the reflex activity of the motor nerve cells of the cord, we may simplify our analysis of these reflex phenomena in their relation to disease. We shall first consider the abnormal conditions that result in decrease or abolition of reflex action.

In the first place, we may have decrease or abolition of the reflexes, through impairment of the peripheral terminations, either of the motor or sensory nerves. Such peripheral motor disturbance is best illustrated by the simple rheumatic palsies, the affected muscle being unable to respond to the motor impulse. A less familiar instance of impairment of the sensory nerve endings occurs in pseudo-muscular hypertrophy, probably by reason of disease in the interstitial muscular tissue in which the afferent nerve begins.

Secondly, we may have interruption to nerve excitation, and loss of the reflexes, somewhere in the course of a nerve trunk, bring to a level, involving both the motor and sensory nerve fibres. Such local palsy might result from a blow, from pressure upon, from degeneration or section of, the nerve trunk. As familiar examples of this class, we have that form of facial paralysis due to pressure upon the nerve in its course through the fallopian canal; and musculo-spinal paralysis, often produced during prolonged sleep, or a drunken stupor, from lying with the arm under the body.

Thirdly, we may have absence of the reflex from interruption to nerve irritability in the course of the posterior-sensory nerve roots; or of the anterior-motor nerve roots, above the point of division of a mixed nerve; as in certain stages of chronic meningitis from pressure.

Fourthly, the tendon reflexes, but not always the skin reflexes, are abolished in sclerosis or degeneration of the posterior columns of the cord; the essential pathological condition of that disease, known as locomotor ataxia.

The other morbid condition in which we note an absence of the tendon reflex is disease of the gray matter, especially the anterior columns of nerve cells, that are supposed to preside over the transformation of sensory impressions into motor impulses. Such degeneration we get in infantile paralysis and allied diseases. In diphtheritic paralysis, we get loss of the tendon reflex also, the gray matter probaby being involved in the morbid process.

On the other hand, whenever we have increased irritability of the reflex loop in any portion, as a consequence we shall get exaggerated myotatic contractions. Thus, the early stages of neuritis and the action of certain poisons on the nerve endings furnish examples of increased irritability in the peripheral portion of the reflex loop. Often, indeed, the exaggerated irritability of the sensory or centripetal tract is so great that spasms form its reflex motor expression. In this class are included the convulsions or spasms due to visceral disorders in general. Traumatic injuries to the nerves, neuralgias, hyperæsthesia, and muscular inflammation may be enumerated as peripheral causes of exaggerated reflex action.

Central causes for exaggeration of the reflexes are very limited, inasmuch as disease of the spinal cord, or its envelopes, is so soon followed by paralysis and abolition of the reflex function. But in certain stages of myelitis, and perhaps in the very beginning of acute meningitis, we get exaggerated reflex phenomena. In the several forms of myelitis especially, do we see the various grades of reflex irritability, from intense spasms to entire absence of nerve re-action. The persistence or marked increase of reflex activity is of great importance in determining the extent and location of the inflammatory process in this disease. The more nearly normal the reflex activity, the more nearly intact will be the gray matter of the cord. If, however, we have a transverse myelitis above the lumbar enlargement, there will be increased irritability in the centers below, manifested by marked knee jerk, ankle clonus, and other forms of exaggerated myotatic contractions, due to the interruption of inhibitory action, as will be shown later. Indeed, the ankle clonus and augmented patellar reflex are best studied under the effects of a myelitis, produced by slow compression of the cord high up.

This brings us to another important class of diseases that cause overaction of the reflex function. In describing the anatomy of the cord, I alluded to the lateral columns as the probable paths between the motor ganglia of the cord and the inhibitory centers in the brain, through which a normal restraint was exerted over the reflex activity of the spinal motor ganglia. It will be clear that whatever destroys or obstructs these paths of conduction, will interrupt this inhibitory action. As a consequence, the motor ganglia below the point of such interruption will re-act more readily, showing again increased irritability. This is illustrated in the instance of transverse myelitis referred to above. But as we often get slow degeneration or sclerosis of the posterior columns, manifested in locomotor ataxia, so we may also have, without the intervention of acute inflammation, degeneration or sclerosis of the lateral conducting columns, the external manifestation of such condition being that disease known as spasmodic paraplegia.

In this malady we have all the phenomena of exaggerated tendinous reflexes, greatly increased knee jerk and ankle clonus, as well as a well-marked peroneal clonus. In pronounced cases it is only necessary to rest the weight of the limb upon the heel to establish a characteristic ankle clonus; and I well remember a case under my observation, in which tapping of the patellar tendon, at short, regular intervals excited such persistent contraction of the quadriceps femoris, that the leg was sustained nearly in complete extension. Indeed, it is supposed that exaggerated reflex activity is the cause of the characteristic spasmodic gait, mere contact with the floor establishing abnormal muscular tension and producing a stiff, jerky muscle action. The impairment of co-ordination so frequently met with in spinal maladies may be partially explained by this alteration in the normal conditions of reflex activity.

In recent simple hemiplegia from brain disease, there is only slight impairment of the functional activity of the cord, and, as a consequence, very little impairment of the normal reflex activity. But often, in the later stages and in some complicated forms of this trouble, the lateral columns high up become secondarily affected with a descending degeneration, and all the phenomena of increased irritability—from cutting off the inhibitory action—will be established, just as in the primary conditions I have recounted. This cause, however, will be distinguished from primary disease of the lateral columns by the existence of a precedent hemiplegia, as well as by other symptoms. Indeed, disease in any part of the cord as well may give rise to a degenerative process, descending along the lateral columns, perhaps, by reason of the longitudinal and oblique conducting fibres, and thus producing the characteristic symptoms.

This paper was not written with any thought of adding to the knowledge of the subject already established, but rather with the hope of rendering such knowledge more serviceable, nor does it aim to be a minute analysis of reflex phenomena and their relation to all morbid states of the spinal cord.

The conditions giving rise to convulsions or spasms, either local or general, depending doubtless to a great degree upon reflex processes, offer a wide field for careful study and observation, while there are other obscure symptoms of disease, in general more or less subject to reflex action, that would well repay investigation with reference to such relationship.

